# Interaction between AhR and HIF-1 signaling pathways mediated by ARNT/HIF-1β

**DOI:** 10.1186/s40360-022-00564-8

**Published:** 2022-04-26

**Authors:** Mengdi Zhang, Yuxia Hu, Fan Yang, Jingwen Zhang, Jianxin Zhang, Wanjia Yu, Minjie Wang, Xiaoli Lv, Jun Li, Tuya Bai, Fuhou Chang

**Affiliations:** 1 Department of Pharmacy Experimental Teaching Center of Pharmaceutical College, Inner Mongolia Medical University, Hohhot, China; 2 Inner Mongolia Research Center for Drug Screening, Hohhot, China; 3 The Center for New Drug Safety Evaluation and Research of Inner Mongolia Medical University, Hohhot, China; 4 School of Pharmaceutical Science, Shanxi Medical University, Hohhot, China; 5 Department of Pharmacology of Pharmaceutical College, Inner Mongolia Medical University, Hohhot, China; 6 Department of Pharmacology of Basic medical College, Inner Mongolia Medical university, Hohhot, China

**Keywords:** Aryl hydrocarbon receptor, Hypoxia-inducible factor-1α, Aryl hydrocarbon receptor nuclear translocator, CoCl_2_, Benzopyrene

## Abstract

**Background:**

The main causes of lung cancer are smoking, environmental pollution and genetic susceptibility. It is an indisputable fact that PAHs are related to lung cancer, and benzo(a) pyrene is a representative of PAHs. The purpose of the current investigation was to investigate the interaction between AhR and HIF-1 signaling pathways in A549 cells, which provide some experimental basis for scientists to find drugs that block AhR and HIF-1 signaling pathway to prevent and treat cancer.

**Methods:**

This project adopts the CYP1A1 signaling pathways and the expression of CYP1B1 is expressed as a measure of AhR strength index. The expression of VEGF and CAIX volume as a measure of the strength of the signal path HIF-1 indicators. Through the construction of plasmid vector, fluorescence resonance energy transfer, real-time quantitative PCR, western blotting and immunoprecipitation, the interaction between AhR signaling pathway and HIF-1 signaling pathway was observed.

**Results:**

BaP can enhance the binding ability of HIF-1α protein to HIF-1β/ARNT in a dose-dependent manner without CoCl_2_. However, the binding ability of AhR protein to HIF-1β/ARNT is inhibited by HIF-1α signaling pathway in a dose-dependent manner with CoCl_2_.

**Conclusion:**

It is shown that activation of the AhR signaling pathway does not inhibit the HIF-1α signaling pathway, but activation of the HIF-1α signaling pathway inhibits the AhR signaling pathway.

**Supplementary Information:**

The online version contains supplementary material available at 10.1186/s40360-022-00564-8.

## Background

Benzopyrene (BaP) is one of the most serious air pollution and food safety issues worldwide. Exposure to certain chemicals or mixtures of chemicals, including chimney soot, shale oil, vinyl chloride, benzene, and cigarette smoke, is associated with the induction of cancer in humans [1, 2]. These chemicals initiate a complex series of events; in the earliest stage, these chemicals are metabolized into reactive derivatives that can damage DNA and subsequently induce mutations [3, 4]. BaP is present in coal tar, which is found in automobile exhaust, smoke from tobacco, burning wood, and charcoal-grilled foods (such as grilled meat) [5].

Exposure to BaP plays a role in lung carcinogenesis, including by directly inducing biological effects associated with cancer by binding to cytoplasmic receptors (Aryl hydrocarbon receptor, AhR) [6, 7]. AhR is a ligand-dependent transcription factor whose structure includes an aromatic hydrocarbon receptor with a nuclear transfer factor (AhR nuclear translocator, ARNT) domain structure that is similar to the basic helix-loop-helix (bHLH)-Per-Arnt-Sim (PAS) structure [8]. ARNT beta is often referred to as hypoxia-inducible factor (HIF)-1β. The main ligands of AhR include BaP and TCDD [9]. In the absence agonists, such as polycyclic aromatic hydrocarbons, most AhR- and immune avidin-related proteins form complex in the cytoplasm [10]. In the presence of polycyclic aromatic hydrocarbons such as BaP, AhR binds to BaP in the cytoplasm and transfers to the nucleus to form a dimer with ARNT [11, 12]. One of the hallmarks of AhR signal transmission activation is the expression of phase I and II genes, such as glutathione-*S*-transferase and cytochrome P450 enzyme, including CYP1A1 and CYP1B1, among others [13]. AhR is transferred to the nucleus and induces the expression of many downstream genes containing the 5′-tngcgtg-3′ sequence of the heterogenous biological response component. These enzymes are thought to be involved in detoxification of heterogenous metabolism and have many biological and toxicological effects by inducing AhR-mediated transcription [14].

ARNT is also known as HIF-1β and can form a dimer with HIF-1α to form HIF-1 and the AhR: ARNT complex. HIF-1α is a major member of the class I bHLH/PAS protein family and known as a crucial oxygen sensor within organisms. HiF-1α regulates and balances aerobic metabolism and energy production to maintain proper oxygen homeostasis. Unlike the ubiquitously expressed ARNT, the presence of HIF-1α depends on the intracellular oxygen concentration, as it is targeted for rapid ubiquitination and proteasomal degradation under normal oxygen conditions [15]. However, HIF-1α is stabilized under conditions of reduced oxygen availability, allowing it to translocate to the nucleus where it binds ARNT, inducing the expression of genes harboring a (5′–G/ACGTG–3′) motif, named as the hypoxia response element [16]. Impaired oxygen homeostasis is common in numerous solid tumors, such as lung cancer. Additionally, the expression of many downstream hypoxia-responsive genes is increased, such as that of vascular endothelial growth factor (VEGF) and carbonic anhydrase-IX (CAIX) [17–19]. Additionally, early in vivo and in vitro studies identified hypoxia as a prerequisite intracellular microenvironment for pulmonary tumorigenesis closely related to chemotherapeutic resistance [20, 21].

Given that ARNT is a shared dimerization partner of both the AhR and HIF-1α signaling pathways, their cellular biological behavior and possibility for crosstalk have been widely examined to understand the interactions between these two signaling pathways. Previous studies revealed a signaling node between the AhR and HIF-1α signaling pathways. However, the antagonistic competition for ARNT recruitment between AhR and HIF-1α is remains controversial. Chan et al., Nie et al., Schults et al., and Lee et al. showed that the HIF-1 and AhR pathways were mutually inhibited [22–24]. In contrast, Gradin et al., Gassmann et al., and others showed that activation of the HIF-1 signaling pathway inhibited the AhR signaling pathway, while the activated AhR signaling pathway did not inhibit HIF-1 signaling pathway [25, 26]. However, no studies have evaluated the physiological, pharmacological, or toxicological aspects of these interactions. Additionally, most studies have focused on liver cancer cells (e.g. Hepa-1 cells, B-1 cells), cervical carcinoma cells (e.g. HeLa cells), and zebrafish live cells (e.g. PLHC-1 cells), etc., while few studies have evaluated human lung cancer cells. The human respiratory system is continuously exposed to various carcinogens that are exogenous ligands for AhR, and hypoxia strongly impacts lung tumorigenesis and drug resistance. Therefore, it is important to clarify the mutual effects between the two important environmental sensing pathways in the context of lung cancer cells.

This study was conducted to analyze the effects of BaP on ARNT/HIF-1β-mediated AhR and HIF-1 signaling pathways with or without CoCl_2_. Additionally, the interaction between the two signaling pathways was clarified. The goal of this study was to improve the understanding of the carcinogenic mechanism of BaP, clarify the interaction between AhR and HIF-1 signaling pathways, and provide an experimental basis for evaluating drugs to block the AhR and HIF-1 signaling pathways to prevent and treat cancer.

## Methods

### Materials

The chemicals used were purchased from the following companies: RPMI-1640 medium (Hyclone, Logan, UT, USA), fetal bovine serum (Gibco, Grand Island, NY, USA), HIF-1α stabilizing regent CoCl_2_ and AhR ligand BaP (Sigma-Aldrich, St. Louis, MO, USA), pDsRed-Monomer-N1 expression vector and pAcGFP1-N1 expression vector (Clontech, Mountain View, CA, USA), Revert Aid First Strand cDNA synthesis kit (Thermo Fisher Scientific, Waltham, MA, USA, Cat. K1621), BglII, KpnI enzyme (Takara, Shiga, Japan), Hoechst 33342(Thermo Fisher Scientific, Waltham, MA, USA, Cat. 1022) CAIX antibody (Santa Cruz Biotechnology, Dallas, TX, USA, Cat. sc-365,900), CYP1B1 antibody (Abcam, Cambridge, UK, Cat. ab33586), CYP1A1 antibody (Abcam, Cambridge, UK, Cat. ab3568), VEGF antibody (Abcam, Cambridge, UK, Cat. ab32152), HIF-1α antibody (Abcam, Cambridge, UK, Cat. ab1), and AhR antibody (Abcam, Cambridge, UK, Cat. ab84833), HIF-1β/ARNT antibody (Cell Signaling Technology, Danvers, MA, USA, Cat. 3781S).

### Methods

#### Cell culture and chemical exposure

Human epithelial lung cancer (A549) cells were purchased from the Chinese Peking Union Medical College Basic Medical Institute (Peking, China). A549 cells were cultured in Dulbecco’s Modified Eagle Medium supplemented with 10% fetal bovine serum at 37 °C in a humidified atmosphere with 20% O_2_ and 5% CO_2_. CoCl_2_ and BaP working solutions (0, 2, 4, 8 μM) were freshly prepared prior to use by dissolving CoCl_2_ and BaP in dimethyl sulfoxide. The cells were seeded at a density of 1 × 10^6^ cells/well in 96-well plates. The treatment dosage and time of CoCl_2_ and BaP were determined by MTT assay and quantitative real-time fluorescence PCR. Appropriate chemical exposure dosages were defined as those that potently stimulated the AhR or HIF-1 signaling pathways to induce downstream gene expression((Xue et al., 2016; Lee et al., 2018).

#### RNA preparation and quantitative real-time fluorescence PCR

Total RNA was extracted from the treated cells using a Total RNA Kit (Tiangen, Beijing, China). The purity of the isolated RNA was determined using a Nano-Drop 2000 (Thermo Fisher Scientific). The A260/280 ratio for each RNA sample was greater than 1.8, and the A260/230 ratio was greater than 2.2. Single-strand cDNA was synthesized from 1 μg total RNA using the Revert Aid First Strand cDNA synthesis kit according to the manufacturer’s instruction.

Real-time PCR was performed to examine the gene expression levels of CYP1A1, CYP1B1, VEGF, CAIX, and β-actin using the following primers obtained from Sangon Biotech (Shanghai, China) (Table 1). qPCR was performed using 1 μL diluted cDNA template in a total volume of 10 μL containing 0.5 μL primer, 5 μL Green PCR Master Mix, and 3 μL RNA-free water. PCR amplification was performed using the following thermocycling program: incubation at 95 °C for 5 min, followed by 40 cycles of 10 s at 95 °C, 30 s at 60 °C, and 30 s at 72 °C.

**Table 1:** Primer sequences for real-time reverse transcription-PCR and plasmid construction

Gene	Sequence
HIF-1a	
Forward primer	5′-TGACTGTGCACCTACTATGTCACTT-3′
Reverse primer	5′-GGTCAGCTGTGGGTAATCCACTC-3′
CAIX	
Forward primer	5′-GCCGCTACTTCCAATATGAGGG-3′
Reverse primer	5′-AACCAGGGCTAGGATGTCACCA-3′
VEGF	
Forward primer	5′-GAACTTTCTGCTGTCTTGGGTGCAT-3′
Reverse primer	5′-GGTCTGCATTCACATTTGTTGTGCTG-3’
CYP1A1	
Forward primer	5′-ATTGGGCACATGCTGACG-3’
Reverse primer	5′-TGCTGGCTCATCCTTGACAG-3’
CYP1B1	
Forward primer	5′-GCCGCTACTTCCAATATGAGGG-3’
Reverse primer	5′-AACCAGGGCTAGGATGTCACCA-3’
β-Actin	
Forward primer	5′-CACCTTCTACAATGAGCTGCGTGTG-3’
Reverse primer	5′-ATAGCACAGCCTGGATAGCAACGTAC-3’
AhR	
Forward primer	5′-GAAGATCTATGAACAGCAGCAGCGC-3′
Reverse primer	5′-GGGGTACCCCCAGGAATCCACTGGATGTCAA-3’
ARNT	
Forward primer	5′- CCGCTCGAGTTCTGGGGAGTGGCCTTTCTT-3′
Reverse primer	5′-CGGGATCCAATTCTGAAAAGGGGGGAAAC-3′

#### Plasmid construction

Specific primers used to construct the plasmids pAcGFP1-AhR and pDsRed-Monomer-ARNT were designed by Oligo 7.0 Software based on the human AhR sequences (GenBank Accession Number: NM_001621.4) and human ARNT sequences (GenBank Accession Number: NM_001668.3), and synthesized by Sangon Biotech. The PCR cycling conditions were 95 °C for 5 min, followed by 40 cycles consisting of 95 °C for 30 s, 56 °C for 30 s, and 72 °C for 2 min, with a final extension step for 10 min at 72 °C. The products were purified from an agarose gel using a Gel Extraction Kit (QIAGEN, Hilden, Germany). The pAcGFP1 vector was linearized by the endonucleases BglII and KpnI at 37 °C for 4 h. The pDsRed-Monomer vector was linearized by the endonucleases XhoI and BamHI at 37 °C for 4 h. Vectors containing cloned inserts were transformed into *Escherichia coli* DH5a and incubated overnight at 37 °C. Positive clones were identified by kanamycin screening and digestion reactions followed by sequencing.

#### Fluorescence resonant energy transfer analysis

The recombinant plasmid was transfected into A549 cells using Lipofectamine 3000, and transfection was detected by a high content fluorescence imaging system. Additionally, logarithmic phase transfected cell lines treated with different concentrations of BaP (0, 2, 4, and 8 μM) and 1% dimethyl sulfoxide were evaluated after 24 h of culture using a high-content fluorescence imaging system to observe changes in the fluorescence signal intensity. Take attached on 96-well plates, on the other hand, growth in good condition with 8 μM BaP and 300 μM CoCl_2_ handle cells at the same time. Control group cells were exposed to 8 μM BaP for 24 h after, after which fluorescence signal intensity was measured. FRET analysis was performed as described by Xia and Liu [27].

#### Western blotting assay

Equivalent amounts of protein (20–50 μg) extracted from A549 cells with lysis buffer containing protease inhibitor cocktail were separated by 10% sodium dodecyl sulfate polyacrylamide gel electrophoresis, transferred onto a polyvinylidene fluoride membrane (Sigma-Aldrich), and blocked with 5% skimmed milk powder in Tris-buffered saline containing 0.2% Tween 20 (TBST) for 1 h at 27 °C. The membranes were washed four times with TBST buffer and incubated at 4 °C overnight with the appropriate primary rabbit antibodies specific for CAIX (1:200), VEGF (1:1000), CYP1A1 (1:500), and CYP1B1 (1:1000). After four washes with TBST, the immunoblots were incubated for 2 h at room temperature with a secondary antibody conjugated with IRDye®700CW Goat (polyclonal) anti- rabbit IgG (LICOR) or IRDye®800CW Goat (polyclonal) anti- mouse IgG (LICOR). A monoclonal mouse anti-β-actin primary antibody (ZSGB-BIO, Beijing, China) was used as an internal control. Finally, each protein was detected with an Odyssey infrared laser imaging system (LICOR).

#### Co-immunoprecipitation

A549 cells were lysed in TNE buffer (25 mM Tris-HCl (pH 7.5), 150 mM NaCl, 1 mM EDTA, 1% NP-40, 5% glycerol). The lysates were immunoprecipitated with anti-HIF-1β/ARNT antibody (D28F3, Cell Signaling Technology) (1:50) followed by western blotting. Co-immunoprecipitation was conducted to examine the interaction of AhR and HIF-1α using the Pierce® Classic IP Kit (Rockford, IL, USA). Western blotting was performed as described above using mouse anti-HIF-1α monoclonal antibody (Abcam) (1:500) or mouse anti-AhR monoclonal antibody (Abcam) (1:500). Monoclonal mouse anti-β-actin primary antibody was used as an internal control.

#### Statistical analysis

All experiments were performed at least three times and showed similar results. Statistical comparisons were performed using one-way and two-way analysis of variance between the different treatments. The effects of different times and treatments were analyzed by multiple *t*-tests. A value of *P* < 0.05 was regarded to indicate significance.

## Results

### Establishment of cell spent oxygen microenvironment and BaP concentration

**Fig. 1:**
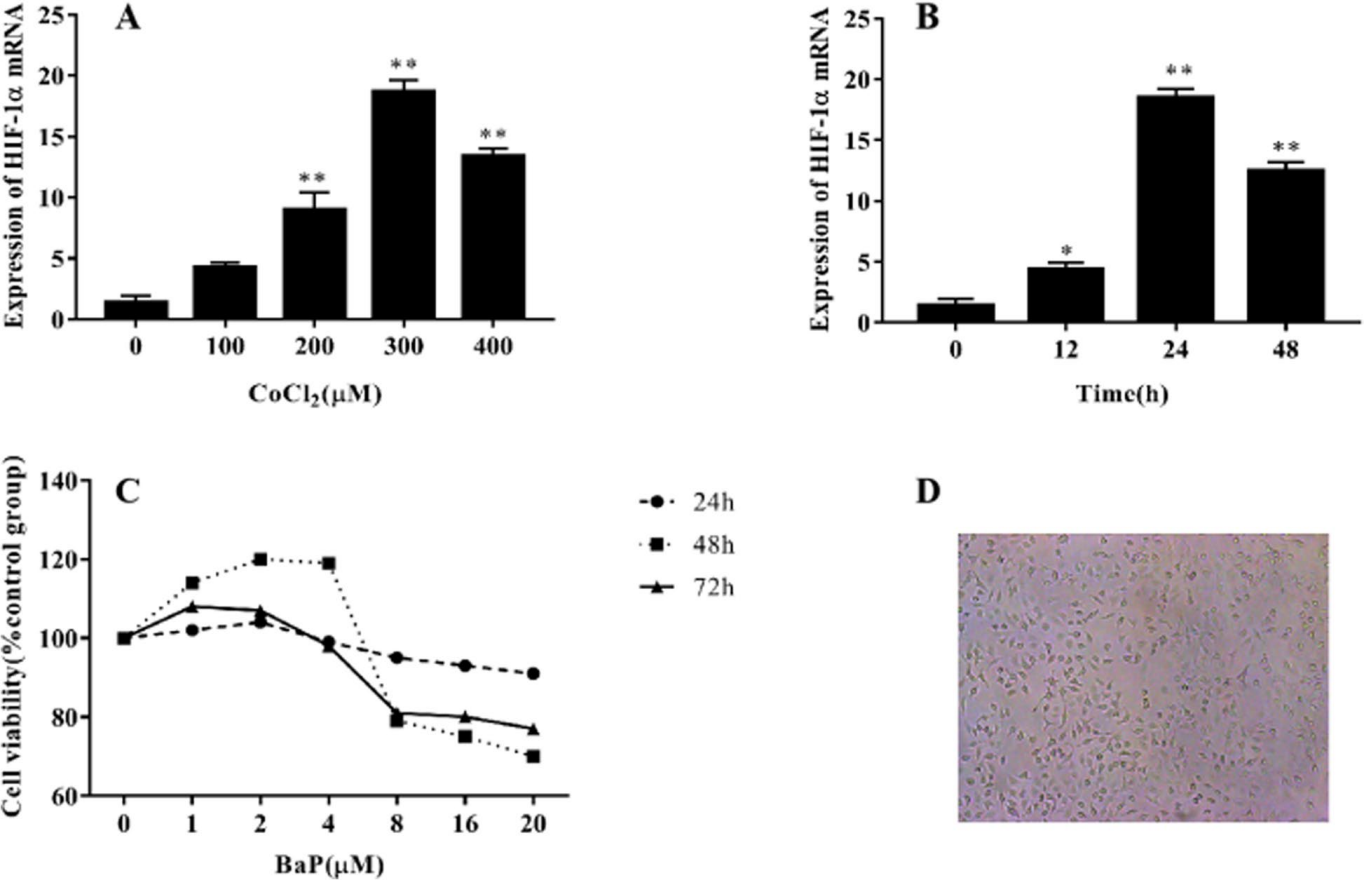
Establishment of cell spent oxygen microenvironment and selection of BaP concentration. **A** Expression level of HIF-1α mRNA after 24 h of treatment with different concentrations of CoCl_2_. **B** Effects of 300 μM CoCl_2_ on expression level of HIF-1α mRNA at different times in A549 cells. **C** Effects of different concentrations BaP on cell viability at different times in A549 cells. **D** A549 cell culture map, *compared to control group *P* < 0.05, **compared to control group *P* < 0.01, *n* = 6

The expression level of HIF-1α mRNA increased with increasing CoCl_2_ concentrations (*P* < 0.05). At a CoCl_2_ concentration of 400 μM, the expression level of HIF-1α mRNA was decreased, indicating that CoCl_2_ promotes the expression of HIF-1α mRNA in a certain concentration range (Fig. 1). For analysis of drug action, 300 μM CoCl_2_ was used.

For CoCl_2_ treatment times of 0, 12, and 24 h, the expression level of HIF-1α mRNA increased over time. The effect of 300 μM CoCl_2_ on A549 cells was significant (*P* < 0.01). After 48 h, the expression level of HIF-1α mRNA decreased, indicating that the anaerobic environment caused by CoCl_2_ promoted HIF-1α mRNA expression within a certain time range (Fig. 1). Therefore, a treatment time of 24 h was used to simulate a hypoxia mimicking conditions.

The effect of different concentrations of BaP at 24, 48, and 72 h in A549 cells was evaluated by MTT assay. The results showed that a lower concentration of BaP was not toxic towards cells and significantly promoted the proliferation of A549 cells (*P* < 0.05). Therefore, BaP concentrations of 0, 2, 4, and 8 μM were used to evaluate the drug action mechanism at 24 h (Fig. 1).

### Plasmid construction

**Fig. 2:**
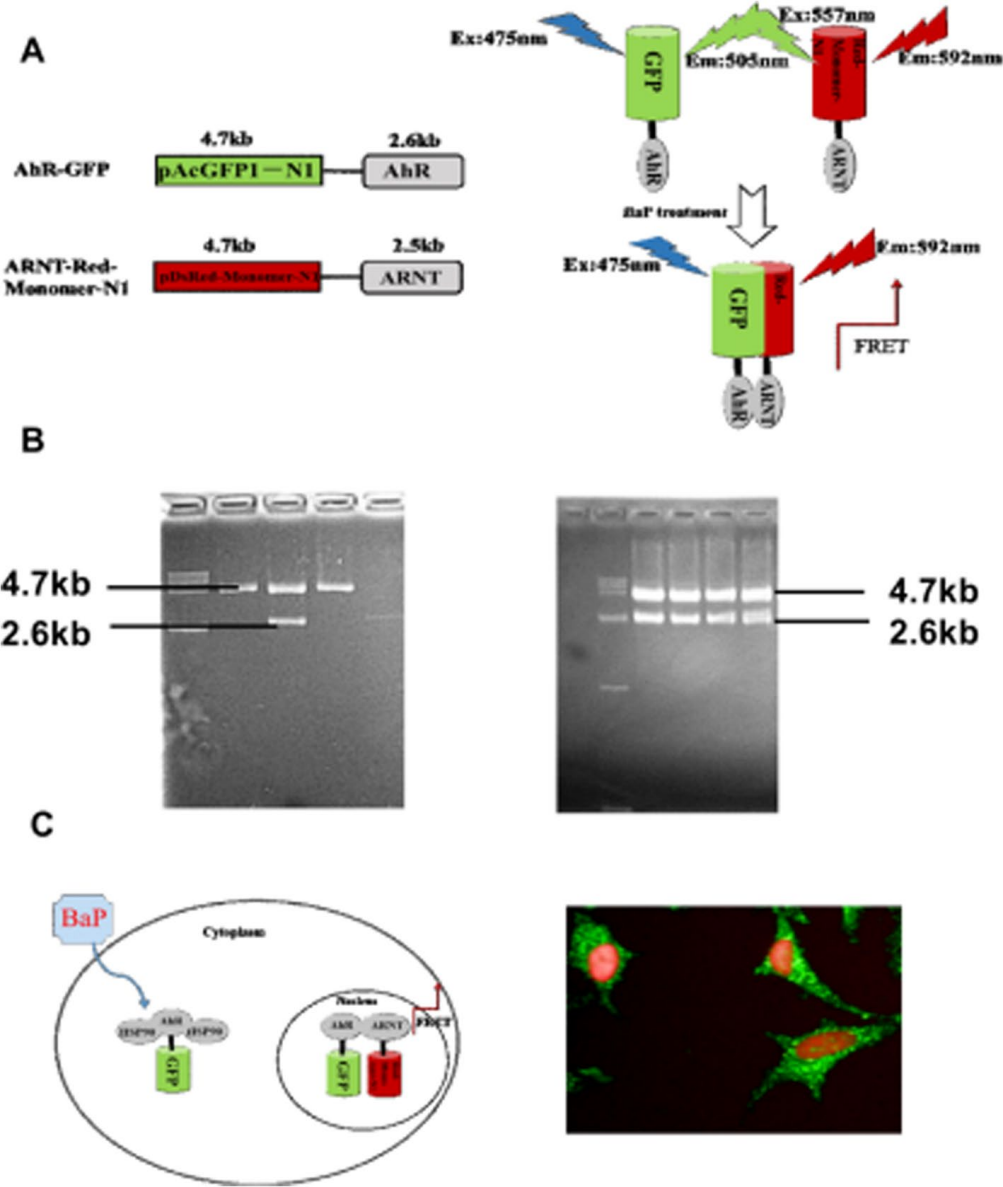
Schematic diagram of eukaryotic expression vector. **A** Bioassay based on FRET. **B** pAcGFP1-AhR and pDsRed-Monomer-ARNT enzyme-digested results of agarose gel electrophoresis. **C** Recombinant plasmid transfection

According to sequencing analysis, the plasmids pAcGFP1-AhR and pDsRed-Monomer-ARNT were constructed in the proper sites and the sequence was correct. We also observed green fluorescent signals in the cytoplasm and red fluorescent signals in the nucleus, indicating that the proteins in the plasmids were successfully expressed in A549 cells (Fig. 2).

### Exposure to BaP induces FRET signals in A549 cells

**Fig. 3:**
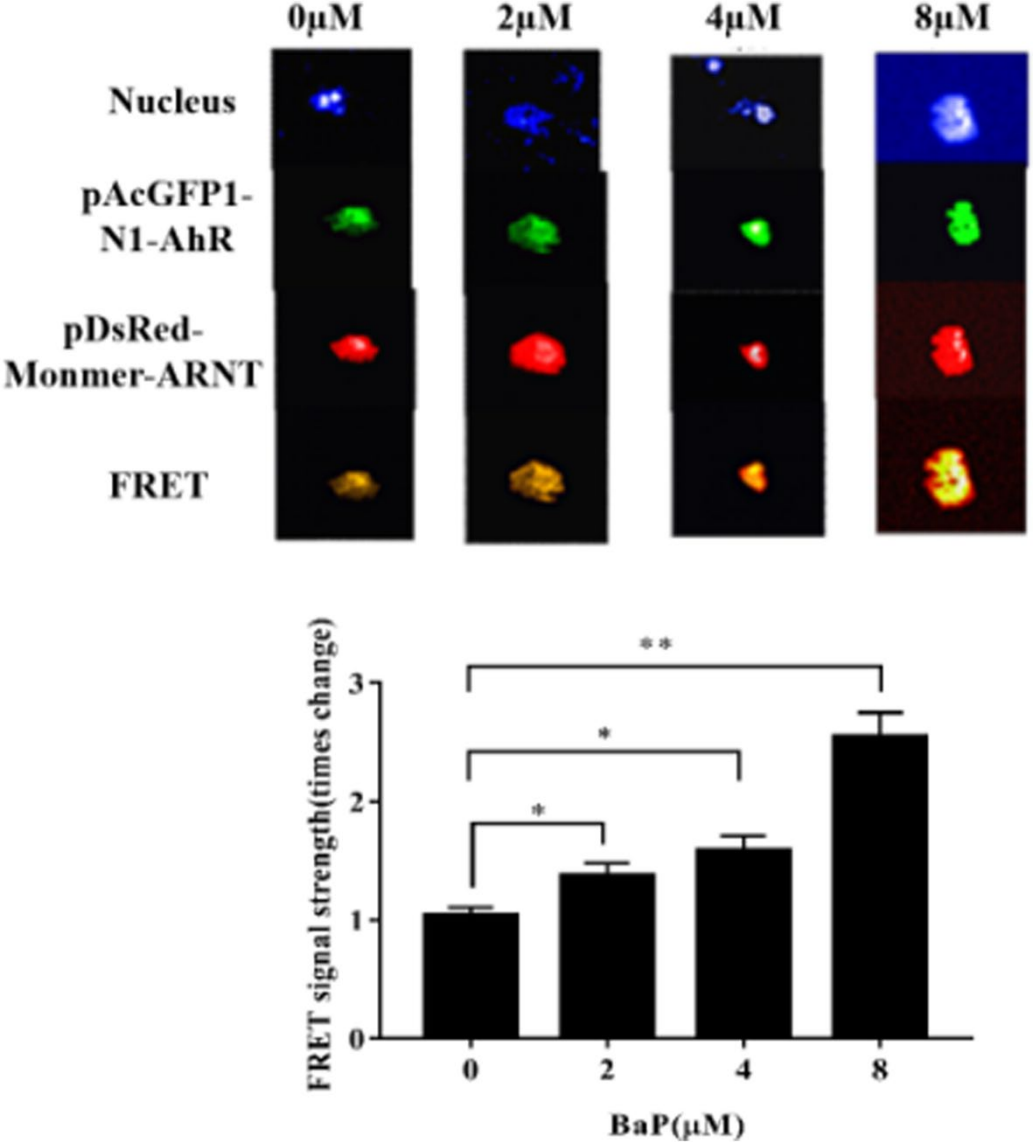
BaP in A549 cells enhanced the FRET fluorescence signal of pAcGFP1-N1-AhR and pDsRed-Monomer-ARNT. **A** pAcGFP1-N1-AhR and pDsRed-Monomer-ARNT recombinant plasmids were transfected into A5F49 cells. After 24 h, the cells were treated with 0, 2, 4, and 8 μM BaP, and then the following channels were used for high content detection: nucleus, donor: AhR, receptor: ARNT and FRET. **B** Bar chart showing changes in the ratios of FRET signal strength,* *P* < 0.05, ** *P* < 0.01, *n* = 3

Co-transfection of pAcGFP1-AhR and pDsRed-Monomer-ARNT recombinant plasmid in A549 cells was performed, followed by exposure to BaP, and then FRET signals were evaluated. After 24 h transfection, the A549 cells were treated with different concentrations of BaP (Fig. 3). According to the statistical analysis of the fluorescence signal, as the concentration of BaP was increased, the intensity of the FRET fluorescence increased. These data suggest that BaP promotes translocation of AhR into the nucleus to form an isodimer with ARNT, further indicating that BaP promotes activation of the AhR signaling pathway.

### Exposure to BaP induces FRET signals in A549 cells under CoCl_2_ conditions

**Fig. 4:**
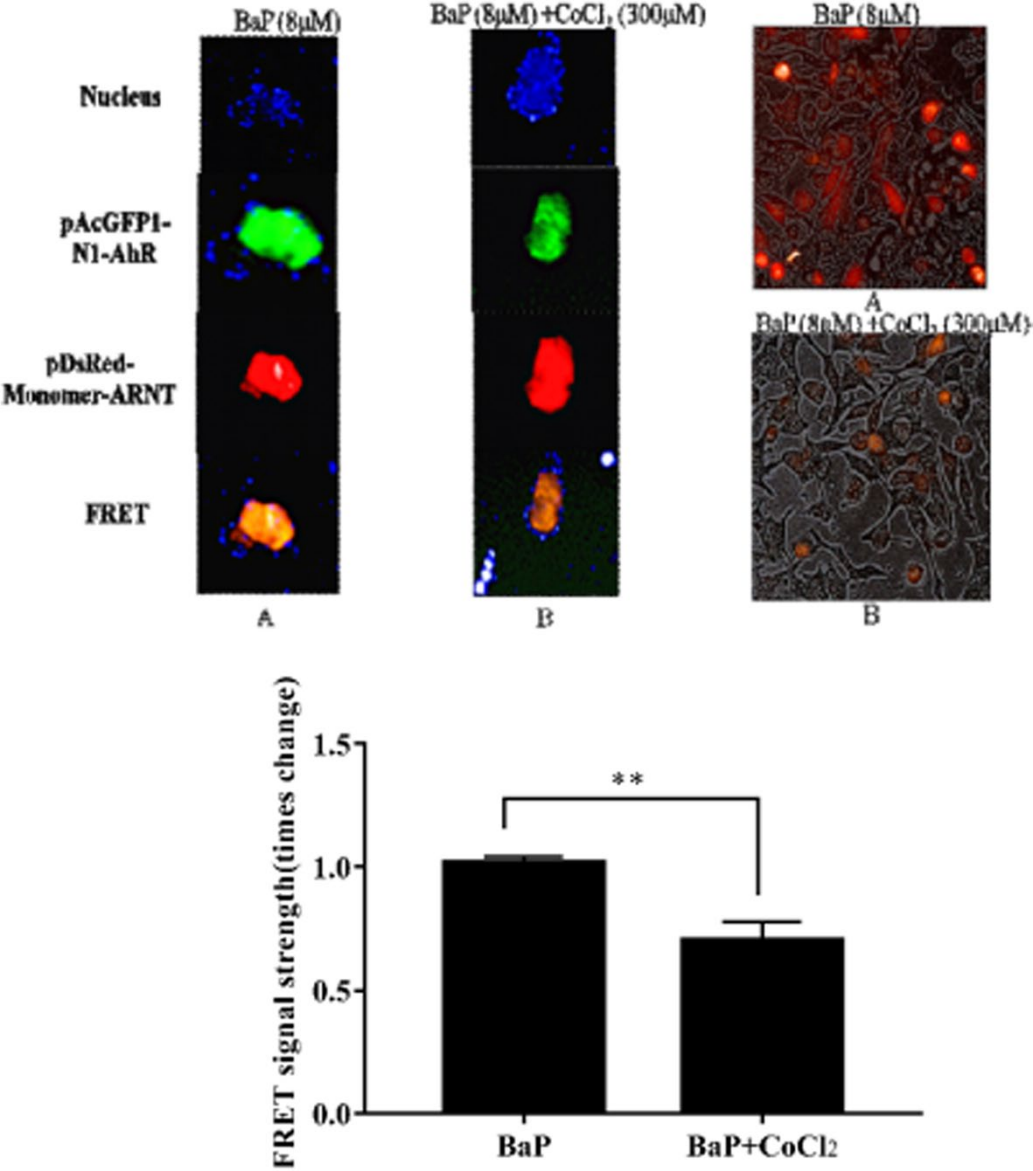
CoCl_2_ in A549 cells reduced the FRET fluorescence signal of pAcGFP1-AhR and pDsRed-Monomer-ARNT. **A** pAcGFP1-N1-AhR and pDsRed-Monomer-ARNT recombinant plasmids were transfected into A549 cells; 24 h later, the cells were treated with 8 μM BaP for 24 h. **B** pAcGFP1-AhR and pDsRed-Monomer-ARNT recombinant plasmids were transfected into A549 cells; 24 h later, the cells were treated for 24 h with 8 μM BaP under hypoxic conditions. In A549 cells, BaP enhanced the pAcGFP1-AhR and pDsRed-Monomer-ARNT FRET fluorescent signal in dose-dependent manner,* *P* < 0.05, ** *P* < 0.01, *n* = 3

We further evaluated the changes in the FRET fluorescence signal intensity with or without CoCl_2_, as BaP significantly enhanced the FRET signal in A549 cells. The FRET fluorescence signal under CoCl_2_ conditions in the recombinant plasmid transfection cell line was weaker than that without CoCl_2_ (Fig. 4). This suggests that HIF-1α binding to ARNT inhibited the binding of AhR and ARNT.

### Effects of BaP on CAIX, VEGF, CYP1A1 and CYP1B1 mRNA expression with or without CoCl_2_

**Fig. 5:**
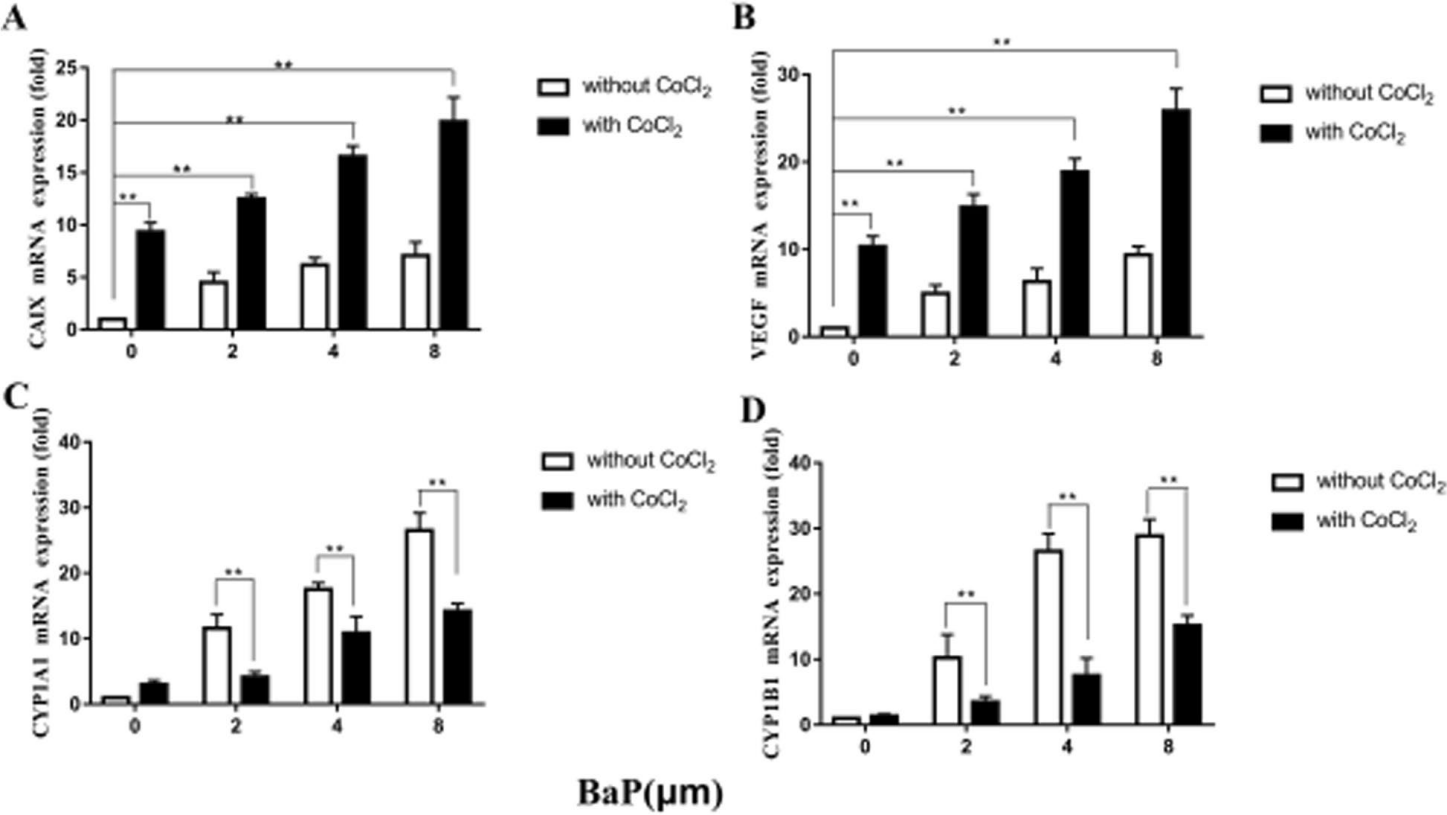
Effects of BaP on CAIX (**A**), VEGF (**B**), CYP1A1 (**C**), and CYP1B1 (**D**) expression at the mRNA level without (□) or with (■) CoCl_2_ and with 0–8 μM BaP. Data are presented as the fold-increase compared to without B(α) P and CoCl_2_, * *P* < 0.05, ** *P* < 0.01. Without (□) or with (■) CoCl_2_ and with 0–8 μM BaP. Data are presented as the fold-increase compared to without BaP and CoCl_2_, * *P* < 0.05, ** *P* < 0.01, *n* = 3

The effect of BaP on the HIF-1 pathway was examined by determining changes in the mRNA of CAIX and VEGF. A549 cells were exposed to different concentrations of BaP in a hypoxia mimicking conditions. CAIX and VEGF expression at the mRNA level was increased compared to cells without CoCl_2_. After adding BaP in the hypoxia mimicking conditions, the mRNA expression of CAIX and VEGF increased gradually with increasing BaP concentrations (Fig. 5).

**Fig. 6:**
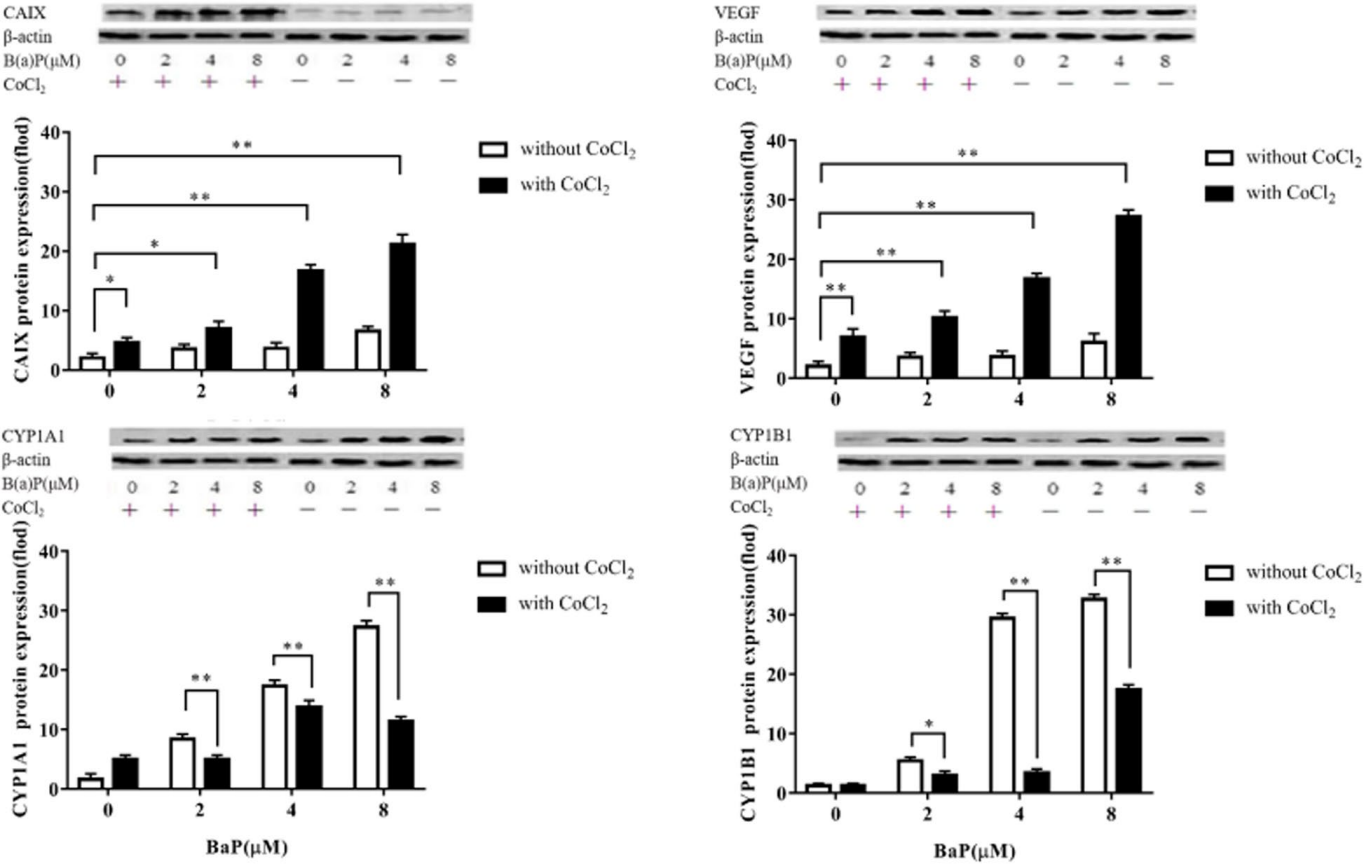
Effects of BaP on CAIX, VEGF, CYP1A1 and CYP1B1 expression at the protein level without (□) or with (■) CoCl_2_ and with 0–8 μM BaP. Data are presented as the fold-increase compared to without B(α) P and CoCl_2_, * *P* < 0.05, ** *P* < 0.01. Without (□) or with (■) CoCl_2_ and with 0–8 μM BaP. Data are presented as the fold-increase compared to without B(α) P and CoCl_2_, * *P* < 0.05, ** *P* < 0.01, *n* = 3

To determine the influence of induction of the HIF-1 pathway on the AhR pathway, the mRNA levels of CYP1A1 and CYP1B1 were investigated in a hypoxia mimicking conditions. As a result, BaP caused a dose-dependent increase in both CYP1A1 and CYP1B1 expression at the mRNA level. After adding BaP in the hypoxia mimicking conditions, this dose-dependent increase in CYP1A1 mRNA levels was significantly down-regulated (*P*<0.01) (Fig. 6).

### Effects of BaP on CAIX, VEGF, CYP1A1 and CYP1B1 protein expression with or without CoCl_2_

Protein expression was evaluated in the same manner. The results showed that as the concentration of BaP increased in the hypoxia mimicking conditions, the protein expression of CAIX and VEGF increased gradually, showing the same dose-dependent trend as the mRNA levels (Fig. 6).

The same conditions were used to test the effect of hypoxic conditions on CYP1A1 and CYP1B1 protein expression. The results were consistent with those of mRNA analysis. BaP up-regulated CYP1A1 and CYP1B1 protein expression levels. When the cells were exposed to both BaP and CoCl_2_, CYP1A1 and CYP1B1 expression was significantly decreased compared to in cells not exposed to CoCl_2_ (*P*<0.01) (Fig. 6).

### Effects of BaP and CoCl_2_ exposure on HIF-1α, AhR, and ARNT protein-protein interaction

**Fig. 7:**
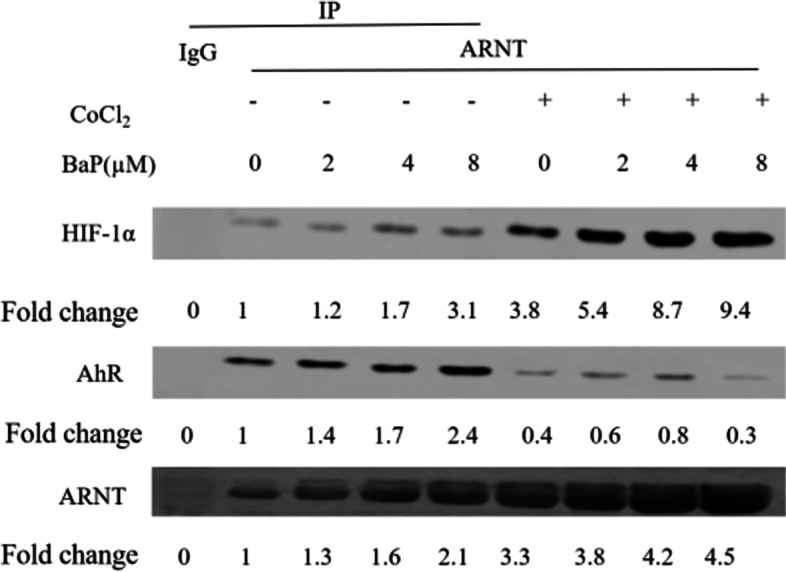
Effects of BaP and CoCl_2_ exposure on HIF-1α, AhR, and ARNT protein-protein interaction. A549 cells were incubated without or with 300 μM CoCl_2_ and with 0–8 μM BaP. Representative western blots show the amount of HIF-1α, AhR bound to ARNT and ARNT bound to AhR. Data are presented as the fold-increase compared to without CoCl_2_ and BaP corrected for the amount of protein loaded, *n* = 3

To directly assess the influence of pathway convergence on protein complex interactions, HIF-1α- and AhR-bound ARNT were immunoprecipitated. As the concentration of BaP increased, the binding of HIF-1a and ARNT increased significantly under CoCl_2_ conditions. AhR binding to ARNT was decreased by 2–8 fold when the HIF-1 pathway was induced compared to in cells without CoCl_2_ (Fig. 7).

## Discussion

In this study, CoCl_2_ activated the HIF-1 signaling pathway. In experiments, two types of hypoxia can be used: environmental hypoxia and hypoxia mimicking conditions. Environmental hypoxia is typically induced in incubation chambers that maintain an oxygen-depleted environment by regulating the gas composition in the chamber [28]. These chambers limit the range of conditions that can be evaluated in an individual study, and the cells quickly establish normoxia when they are removed for manipulation. Hypoxia mimicking conditions is the addition of iron chelating agents or CoCl_2_ to the cells, the purpose of which is to block the transduction of oxygen signals and simulate hypoxia signal transduction in the cells. Co^2+^ is the substrate of iron chelatase, which can replace Fe^2+^ in the oxygen sensor heme, and binds to oxygen at high concentrations to lock this molecule in a deoxygenated state. Its function is to make cells “feel” hypoxia in a non-hypoxic environment, and induce the expression of hypoxia-inducible factor-1 (HIF-1) and its regulatory genes in cells to enhance the anti-hypoxia ability of cells [29]. Because CoCl_2_ mimics hypoxia by stabilizing HIF-1α expression regardless of the oxygen levels, this method is more stable than using conventional hypoxic chambers [30]. Studies have shown that CoCl_2_ does not affect cell culture [31]. Therefore, CoCl_2_ is a better chemical anoxic inducer [32]; we treated A549 cells with CoCl_2_ to simulate chemochemical hypoxia in vitro. Most studies targeting the AhR signaling pathway use TCDD to activate AhR. Although TCDD is a strong activator of AhR, it is not metabolized by the CYP protein induced by the AhR pathway [33]. Therefore, we used BaP as an agonist of AhR.

High-content technology is a high-resolution fluorescent digital imaging system that uses fluorescent methods to mark the target in the cell and obtain clear image information by automatic fluorescence microscopy [34]. Calculation software can be customized for biological analysis to quantitatively analyze the images, obtain multi-parameters result for each cell, and finally output the statistical results related to cell biological phenomenon required by the user. To quantitatively evaluate various environmental factors, various external stimuli, or the effects of various compounds on cells, the experiments were conducted in a closed space to reduce external interference (such as from fluorescent lamps) and reduce errors in the experimental results [35]. In this study, A549 cells were transfected with the recombinant plasmids pAcGFP1-N1-AhR and pDsRed-Monomer-N1-ARNT and changes in the FRET fluorescence signal intensity were detected in the presence of difference concentrations of BaP before and after CoCl_2_. These changes were initially used to investigate the interaction between AhR and HIF-1 proteins. AhR was detected in the cytoplasm, while ARNT was observed in the nucleus; the FRET fluorescent signal was also observed in the nucleus. In the presence of different concentrations of BaP, HIF-1α competed with AhR for binding to ARNT/HIF-1β protein, further indicating that activation of the HIF-1α signaling pathway inhibits the AhR signaling pathway.

Both AhR and HIF-1α proteins are important sensors of environmentally carcinogenic changes; thus, their potential crosstalk has been investigated based on the interaction between the two pathways. Previous studies demonstrated that HIF-1α- and AhR-activated gene expression is mutually or partly inhibited under simultaneous exposure to xenobiotic and hypoxia stress [36]. In our study, AhR-induced gene expression was suppressed under mimicked CoCl_2_ conditions, which agrees with previous studies. Unlike others, activation of the HIF-1 signaling pathway is dose-dependent on the concentration of BaP action. PCR and western blot results reveled that combined BaP and CoCl_2_ significantly decreased CYP1A1 and CYP1B1 expression at both the mRNA and protein levels in A549 cells compared to incubation with BaP alone. In contrast, the expression of VEGF and CAIX within HIF-1 signaling pathway was elevated under the same treatment, indicating that the AhR and HIF-1 signaling pathways do not simply suppress each other. Previous studies showed that compared to AhR, HIF-1α has a greater affinity for ARNT in vitro [25, 37]. We observed that AhR activation did not impair HIF-1α-dependent induction of downstream gene expression while enhancing signaling. To further study this mutual interaction between AhR and HIF-1, we performed immunoprecipitation to determine the amount of HIF-1α and AhR protein bound to ARNT following co-treatment with BaP and CoCl_2_. Levels of the HIF-1α: ARNT complex increased with increasing BaP concentrations and 300 μM CoCl_2_ treatment compared to in cells exposed to only hypoxia stress. These results were confirmed at both the mRNA and protein levels.

Studies of the interaction of AhR and HIF-1 signaling pathways have shown controversial results. Here, we summarize several possible explanations for this phenomenon. First, it may because of the different experimental models used in various studies. In terms of different signaling pathway stimulants, different agents can show disparate biological effects. For example, Li et al. found that benzo [a]pyrene-3,6-dione (BPQ), but not BaP and benzo [a]pyrene-7,8-diol-9,10-epoxide (BPDE), inhibited VEGF expression in a dose-dependent manner [38]. Even under stimulation with the same agonist, BPQ did not inhibit HIF-1α mRNA levels, but inhibited its protein expression in a proteasome-dependent manner. Second, recent studies has challenged the traditional view that ARNT is unchanged at the AhR:ARNT:HIF-1 signaling node. Previously, ARNT was considered as ubiquitously and constitutively expressed, indicating that neither the ARNT mRNA nor protein level is influenced by hypoxia stress [39]. However, emerging evidence has indicated that ARNT is up-regulated under low oxygen conditions in a cell-specific manner [40]. For instance, Zhong et al. observed increased expression of ARNT after CoCl_2_ incubation in PC-3 cells [41]; Mandl et al. showed that ARNT is up-regulated in an HIF-1α-dependent manner in 518A2 human melanoma cells under hypoxic conditions [40]; Kim et al. concluded that AhR and ARNT expression was dose- and time-dependently up-regulated in PCB-, BaP-, and TBT-exposed intertidal copepod *Tigriopus japonicas* [42]. This cell-specific expression of ARNT may explain why the competition between AhR and HIF-1α for ARNT dimerization was inconsistent in different studies using cells derived from different entities. However, the hypothesis that ARNT is elevated under CoCl_2_ in A549 cells and that ARNT has stronger affinity for HIF-1α is plausible, as AhR signaling pathway induction promoted the hypoxia signaling activity induced by CoCl_2_. Finally, other active proteins may be involved in AhR and HIF-1α signaling pathways apart from ARNT; the underlying influences on these factors are not examined when exploring the mutual interaction of the two pathways. A recent study evaluated the mechanism of NcoA2-regulation of the AhR-ARNT-HIF-1α interaction, which may depend on both ARNT and NcoA2. NcoA2 is involved in regulating hypoxia response element and heterogenous biological response component transactivation as well as competes with HIF-1α and AhR to form protein complexes with ARNT [40].

Increasing studies have highlighted the importance of both the AhR and HIF-1α signaling pathways in the development of lung cancer. However, only two studies examined the interaction of the two pathways in lung cancer cells in detail, which showed opposite conclusions as in our study. Schults et al. found that HIF-1α activation attenuated BaP-induced AhR-mediated gene expression [33]. In our study, BaP down-regulated CoCl_2_-mediated induction of CAIX in a dose-dependent manner, suggesting an inhibitory relationship between the two pathways. The differences from our result may be related to the different concentrations of BaP used. We used BaP concentrations from 0 to 8 μM, while Schults et al. treated A549 cells with concentrations from 0 to 1 μM. As studies demonstrated that BaP-induced expression of ARNT is dose-dependent in copepod *T. japonicus*, this may account for the converse result [43]. We also unexpectedly found that the activity of the HIF-1α signaling pathway was promoted by co-stimulation of the AhR signaling pathway by BaP in a dose-dependent manner. However, the specific mechanisms should be evaluated in lung cancer cells. Li et al. found that BPQ inhibited VEGF expression at the transcription level in A549 cells, while the corresponding effect of the HIF-1 signaling pathway induction on AhR signaling was not examined [38]. In terms of the unclear interaction between the AhR and HIF-1α signaling pathways, studies of the detailed molecular mechanisms of the two pathways are underway. Our research provides information on the interaction of the two pathways in lung cancer cells, which has not been widely studied. We clarified the effects of BaP on the ARNT/HIF-1β-mediated AhR and HIF-1 signaling pathways with or without CoCl_2_. These results may be useful in the search for drugs to block the AhR and HIF-1 signaling pathways to prevent and treat cancer and for evaluating the cause and prevention of lung cancer.

## Conclusion

Based on the results of the present study. It is shown that activation of the AhR signaling pathway does not inhibit the HIF-1α signaling pathway, but activation of the HIF-1α signaling pathway inhibits the AhR signaling pathway. This project revealed the influence of BaP on the AhR and HIF-1 signaling pathways mediated by HIF-1β/ARNT, which providing experimental basis for the study of the carcinogenesis mechanism of BaP.

## Supplementary Information

**Additional file 1.** The images of CAIX original blots in Fig. 6. Effects of BaP on CAIX expression at the protein level without or with CoCl_2_ and with 0–8 μM BaP.

**Additional file 2.** The images of VEGF original blots in Fig. 6. Effects of BaP on VEGF expression at the protein level without or with CoCl_2_ and with 0–8 μM BaP.

**Additional file 3.** The images of CYP1A1 original blots in Fig. 6. Effects of BaP on CYP1A1 expression at the protein level without or with CoCl_2_ and with 0–8 μM BaP.

**Additional file 4.** The images of CYP1B1 original blots in Fig. 6. Effects of BaP on CYP1B1 expression at the protein level without or with CoCl_2_ and with 0–8 μM BaP.

**Additional file 5.** The images of HIF-1α original blots in Fig. 7. Effects of BaP and CoCl_2_ exposure on HIF-1α, AhR, and ARNT protein-protein interaction. A549 cells were incubated without or with CoCl_2_ and with 0–8 μM BaP. Representative western blots show the amount of HIF-1α bound to ARNT.

**Additional file 6.** The images of AhR original blots in Fig. 7. Effects of BaP and CoCl_2_ exposure on HIF-1α, AhR, and ARNT protein-protein interaction. A549 cells were incubated without or with CoCl_2_ and with 0–8 μM BaP. Representative western blots show the amount of AhR bound to ARNT.

**Additional file 7.** The images of ARNT original blots in Fig. 7. Effects of BaP and CoCl_2_ exposure on HIF-1α, AhR, and ARNT protein-protein interaction. A549 cells were incubated without or with 300 μM CoCl_2_ and with 0–8 μM BaP. Representative western blots show the amount of ARNT bound to AhR.

## Data Availability

The datasets used and/or analysed during the current study are available from the corresponding author on reasonable request.

## Abbreviations

HIF-1: Hypoxia inducible factor-1
TCDD: Tetrachorodibenzo-p-dioxin
BaP: Benzo(a)pyrene
VEGF: Vascular endothelial growth factor
CAIX: Carbonic anhydrase IX
CYP1A1: Cytochrome P450, family 1, subfamily A, polypeptide 1
CYP1B1: Cytochrome P450, family 1, subfamily B, polypeptide 1
ARNT: Aryl receptor nuclear translocator
AhR: Aryl hydrocarbon receptor
RPMI: Roswell Park Memorial Institute
CoCl_2_: Cobalt (II) chloride hydrate
DMSO: Dimethyl sulfone
MTT: 3-(4,5-dimethylthiahiaz(−z-yl)-3, 5-di-phenytetrazoliumromide
