# Supplementary figures and images for: Interaction between AhR and HIF-1 signaling pathways mediated by ARNT/HIF-1β
Source: BMC Pharmacol Toxicol. 2022 Apr 26;23:26. doi: 10.1186/s40360-022-00564-8 (PMC9044668; doi:10.1186/s40360-022-00564-8)

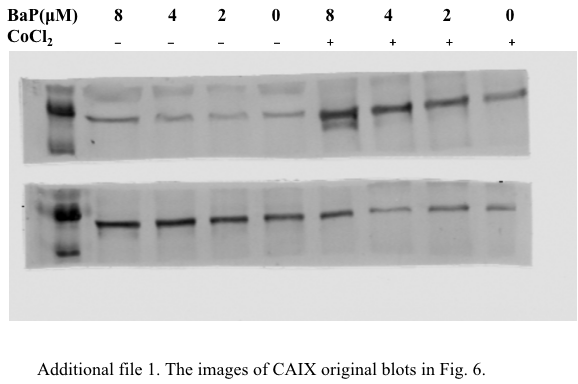

Supplement: Supplementary file 1 — Additional file 1. The images of CAIX original blots in Fig. 6. Effects of BaP on CAIX expression at the protein level without or with CoCl2 and with 0–8 μM BaP. [file 40360_2022_564_MOESM1_ESM.tif]

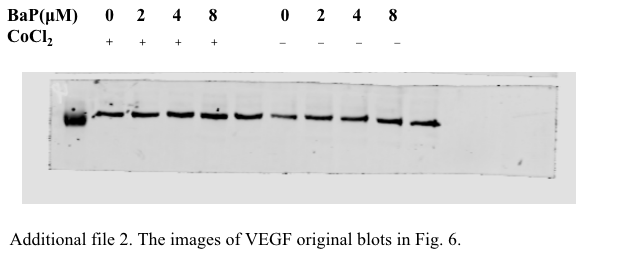

Supplement: Supplementary file 2 — Additional file 2. The images of VEGF original blots in Fig. 6. Effects of BaP on VEGF expression at the protein level without or with CoCl2 and with 0–8 μM BaP. [file 40360_2022_564_MOESM2_ESM.tif]

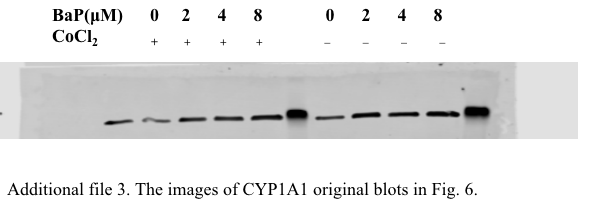

Supplement: Supplementary file 3 — Additional file 3. The images of CYP1A1 original blots in Fig. 6. Effects of BaP on CYP1A1 expression at the protein level without or with CoCl2 and with 0–8 μM BaP. [file 40360_2022_564_MOESM3_ESM.tif]

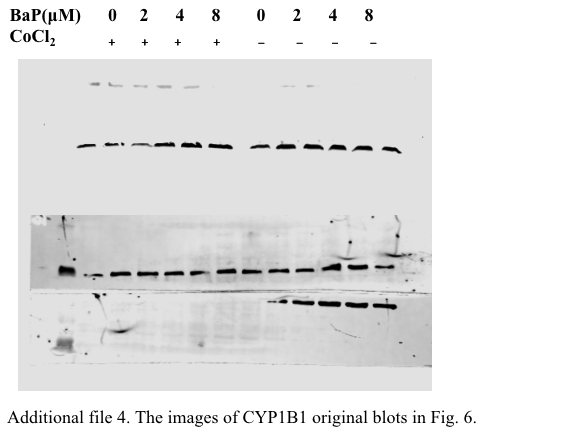

Supplement: Supplementary file 4 — Additional file 4. The images of CYP1B1 original blots in Fig. 6. Effects of BaP on CYP1B1 expression at the protein level without or with CoCl2 and with 0–8 μM BaP. [file 40360_2022_564_MOESM4_ESM.tif]

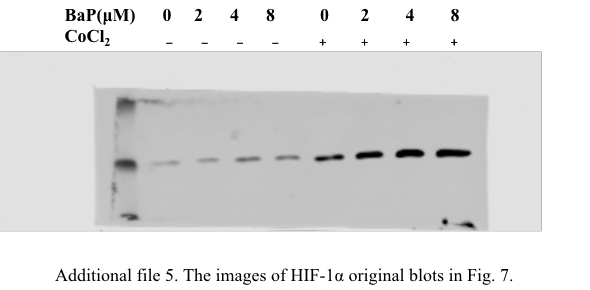

Supplement: Supplementary file 5 — Additional file 5. The images of HIF-1α original blots in Fig. 7. Effects of BaP and CoCl2 exposure on HIF-1α, AhR, and ARNT protein-protein interaction. A549 cells were incubated without or with CoCl2 and with 0–8 μM BaP. Representative western blots show the amount of HIF-1α bound to ARNT. [file 40360_2022_564_MOESM5_ESM.tif]

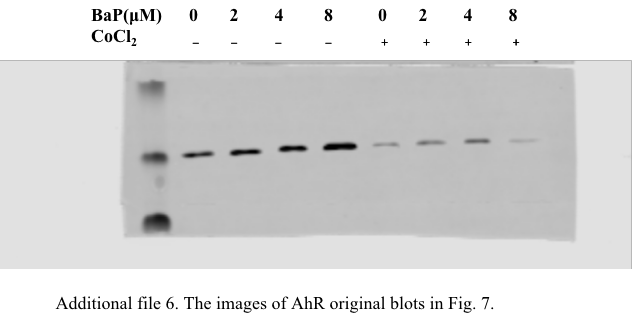

Supplement: Supplementary file 6 — Additional file 6. The images of AhR original blots in Fig. 7. Effects of BaP and CoCl2 exposure on HIF-1α, AhR, and ARNT protein-protein interaction. A549 cells were incubated without or with CoCl2 and with 0–8 μM BaP. Representative western blots show the amount of AhR bound to ARNT. [file 40360_2022_564_MOESM6_ESM.tif]

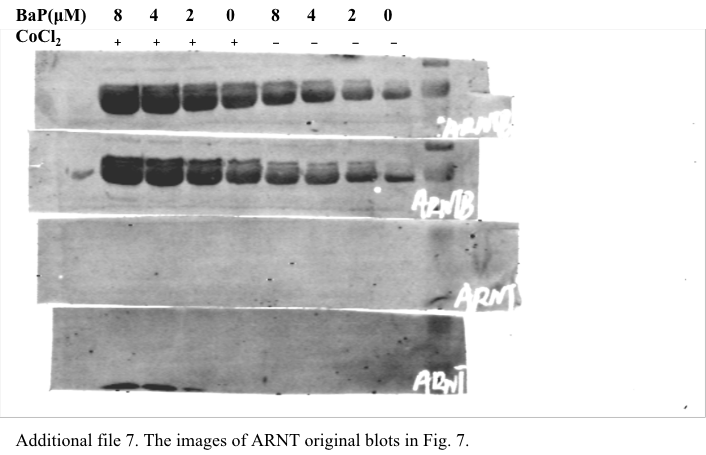

Supplement: Supplementary file 7 — Additional file 7. The images of ARNT original blots in Fig. 7. Effects of BaP and CoCl2 exposure on HIF-1α, AhR, and ARNT protein-protein interaction. A549 cells were incubated without or with 300 μM CoCl2 and with 0–8 μM BaP. Representative western blots show the amount of ARNT bound to AhR. [file 40360_2022_564_MOESM7_ESM.tif]
